# Odontography; or, a Treatise on the Comparative Anatomy of the Teeth, &c.

**Published:** 1846-04

**Authors:** 


MEDICO-CHIRURGICAL

REVIEW.

APRIL, 1846.

ODONTOGRAPHY; OR A TREATISE ON THE COMPARATIVE ANATOMY OF THE TEETH; THEIR PHYSIOLOGICAL RELATIONS, MODE OF DEVELOPMENT, AND MICROSCOPIC STRUCTURE, IN THE VERTEBRATE ANIMALS. By *Richard Owen*, F.R.S. Hunterian Professor to the Royal College of Surgeons of England, &c. 2 Vols. large octavo, pp. lxxiv, 655. Plates 150. Bailliere, 1840-45.

In the midst of the host of compilations, and worse than compilations, that have of late crowded the shelves of our bibliopoles, and swelled their periodical catalogues, it is satisfactory to be able to rest upon a work founded upon sterling observation, and full of the results of great labour, animated by genius and talent. The science of physiology must be taught by monographs. It is, indeed, useful to the tyro to be furnished with those compiled manuals which profess to treat of all the systems of the body in an octavo volume, and do physiology in little—in which the digestive function is fully developed in a short chapter, and twenty pages suffice for the discussion of the whole subject of generation. The young student who seeks only for a text-book by which to guide his initiatory studies may be aided by such means in finding what he has to achieve: the map lies before him, and the main road is pretty distinctly indicated. But it is only in elaborate monographs like that before us that the details of a science can be developed, and any satisfactory acquaintance obtained with its generalities. We have been led to these observations by the character of the extraordinary book which forms the subject of the present article. Its entireness, so to speak, the extent and range of its object, and the completeness of its execution; the great variety of its illustrations, the close observation exhibited in the detail of the facts, the philosophical grandeur of the conclusions deduced from them, and, though last, scarcely least, the profuse number and beauty of the engravings with which the whole subject is illustrated, combine to render this one of the most remarkable works of its day.

In endeavouring to make our readers in some measure acquainted with the scope and objects of this magnificent book, it is not our intention to enter with any polemical spirit into the questions, still controverted, which are discussed in its pages. That there are some theories which appear to us to be scarcely sufficiently supported by the facts and observations detailed, to warrant their being taken as proved, and others which are defended in a tone which the distinguished author might well afford to have modified and softened, takes nought from the real and intrinsic importance of the bulk of the work; and it will be our task rather to introduce it to our readers, in the hope of inducing them to form a personal acquaintance with the whole of its contents, than to debate the *vexatæ questiones*, some of which occur, in limine, on opening the introductory portion of the book.

The scope of the work is co-extensive with the subject itself; and a glance at its plan is sufficient to shew that it must become an essential text-book to the physiologist, to the formal zoologist and to the paleantologist; all of whom are equally interested in its details.

The general structure of the teeth, and the history of their physiology, and the steps by which we have arrived at our present knowledge of their structure, is contained in the Introduction, which extends to upwards of seventy pages, and which may be considered as constituting the more theoretical portion of the work.

The text embraces the most accurate and elaborate descriptions of the number, classification, and uses, as well as of the intimate structure of the teeth of every group of vertebrate animals possessing these organs. It is to Professor Owen that we are thus indebted for the means, most especially important to the palæontologist, of ascertaining by the microscopic structure of the teeth, the true relations of those organic remains which are frequently only fragmentary, and of which, even where the osteology is found in tolerable completeness, the physical characters of the bones are so anomalous, that it is difficult to determine their affinities with known groups:—a principle so important as worthily to rank by the side of that by which the genius of Cuvier first appreciated the relations of the different elements of comparative osteology, and by a bone or the fragment of a bone, seized upon the essential characters of the totality of the extinct animal's organization, and brought up before the mental vision of modern philosophers, the forms of the inhabitants of an ancient world.

The discoveries of many of the earlier physiologists have been sadly overlooked; and it is only of late that a disposition has been manifested of awarding to them their due meed of praise and acknowledgment for what they have really effected. Not a few of the most vaunted discoveries of modern times have been more than glimpsed at by the laborious investigators of past days; and although it would be unfair to suppose that the absence, in many cases, of all reference to their anticipatory discoveries, and of all acknowledgment of their merit, has arisen from any other cause than ignorance of their true claims, yet it would surely be worth a little examination, and the expense of some time and trouble to ascertain, before the enunciation of a presumed discovery, how far it may have been anticipated in past times, were it merely from a sense of justice and a desire of awarding to every one the "suum cuique."

In scarcely any portion of physiological investigation has this ignorance of the discoveries of our predecessors been more remarkable than in the structure of the teeth. From Hunter downwards we have various descriptions of the physical characters of these organs; they are stated by one to be of a bony structure, by another one portion of a tooth is described as organized bone, and another as unorganized enamel,—their "conchoidal fracture," their extreme density, their laminated structure as exhibited in partial calcination, are all described with correctness and accuracy. At length, their true structure is discovered by means of the microscope;—and the "calcigerous tubules" and the fibrous intertubular substance, and the fine enamel fibres, and the intertubular cells, and other points exhibited by their microscopical examination, are all described as new discoveries—and that in perfect good faith—without once recollecting that the essential elements of these structures were suggested by Malpighi, and confirmed by Leeuwenhoek, ages before the modern discoverers were born. It is remarkable that a sufficiently accurate representation and description of the "dentinal tubes," were given by Leeuwenhoek, and that, too, in the Transactions of our own Royal Society, as long ago as 1678; and there it appears to have rested in concealment, within the walls of millboard that covered the venerable volume, unsuspected by the Hunters and Blakes, and their successors; and that even whilst Purkinjé was conceiving and carrying out his admirable investigations, and rediscovering tbe true structure of the teeth with all the means and appliances derived from the superiority of modern instruments, and the advantage of modern science, he was unconscious that one of the most interesting and important of his discoveries had been anticipated by about a century and a half. It is true that Portal formed a remarkable exception to the general ignorance of these early discoverers; and it is the more surprising that such ignorance should have continued, when we find, in the "Histoire de l'Anatomie et de la Chirurgie" of this learned and eminent author, the following passage in reference to the paper in question, and which is quoted by our author.—"Les dents sont composées de très petits tuyaux transparents et droits, dont six ou sept cents égalent à peine, un poil de la barbe." And it is still more remarkable that, notwithstanding the extent of Purkinjé's researches, and the zeal with which he followed them out for a considerable period, it was left to Retzius to direct "the attention of anatomists to Leeuwenhoek's discovery of the structure of Dentine."

The account which the author gives of the intimate structure of the teeth according to our present knowledge of it, is so succinct and comprehensive, that we prefer giving this introductory statement in his own words:—

"True teeth consist of two or more tissues, characterized by the proportions of their earthy and animal constituents, and by the size, form and direction of the cavities in the animal basis which contain the earth, the fluid or the vascular pulp.

"The tissue, which forms the chief part or body of the tooth, has, hitherto, received no distinct and specific name in our language; a particular modification of it, which characterizes the tusks of the elephant, is called 'ivory.' Some Anatomists have extended the application of this term to the analogous substance in all teeth; others have treated of it under the name of the 'bone of the tooth' or 'tooth-bone'; by the German Anatomists it is termed 'knochensubstanz', 'zahnbein' and 'zahnsubstanz'; and some of the latest and most close-thinking writers on dental anatomy have preferred the literal translation of one or other of these terms to the use of the word 'ivory', which unavoidably recalls the idea of the peculiar modification of the 'tooth-substance' in the elephant's tusk, to which it is restricted in common language and in the best zoological works.* I propose to call the substance which forms the main part of all teeth 'dentine.'†

"The second tissue, which is the most exterior in situation, is the 'cement.'‡

"The third tissue, which, when present, is situated between the dentine and cement, is the 'enamel.'§

"'Dentine' consists of an organized animal basis disposed in the form of extremely minute tubes and cells, and of earthy particles: these particles have a two-fold arrangement, being either blended with the animal matter of the interspaces and parietes of the tubes and cells, or contained in a minutely and irregularly granular state in their cavities.

"The density of the dentine arises principally from the proportion of earth in the first of these states of combination; the tubes and cells contain, besides the granular earth, a colourless fluid, probably transuded 'plasma' or 'liquor sanguinis,' and thus relate not only to the mechanical conditions of the tooth, but to the nutrition of the dentine.

"Dentine, thus organized, is 'unvascular': the teeth of most mammals and reptiles, and of a few fishes, present this modification of their main constituent. But the dentine in the teeth of most fishes, of a few mammals, and of still fewer reptiles, is traversed by canals containing blood vessels or a vascular pulp; the tooth-substance, thus modified, I term 'vascular dentine.' Both the 'vascular' and 'unvascular dentine' may be present in the same tooth, as in those of the sloth, the walrus, and the cachalot; the transition from the vascular dentine to true bone is gradual and close.

"'Cement' always closely corresponds in texture with the osseous tissue of the same animal, and wherever it occurs of sufficient thickness, as upon the teeth of the horse, sloth, or ruminants, it is also traversed, like bone, by vascular canals. In reptiles and mammals, in which the animal basis of the bones of the skeleton is excavated by minute radiated cells, forming with their contents the 'corpuscles of Purkinjé,' these are likewise present, of similar size and form, in the 'cement,' and are its chief characteristic as a constituent of the tooth. The hardening material of cement is partly segregated and combined with the parietes of radiated cells and canals, and is partly contained in aggregated grains in the cells, which are thus rendered opake.

"The relative density of the dentine and cement varies according to the proportion of the earthy material, and chiefly of that part which is combined with the animal matter in the walls of the cavities, as compared with the size and number of the cavities themselves. In the complex grinders of the elephant, the masqued boar and capibara, the cement, which forms nearly half the mass of the tooth, wears down sooner than the dentine.

"The 'enamel' is the hardest constituent of a tooth, and consequently the hardest of animal tissues; but it consists, like the other dental substances, of

---

* "The accurate Illiger distinguishes the 'substantia ossea' of a tooth from 'ebur,' and separately defines both these modifications of the tooth-substance. *Prodromus Systematis Mammalium*, 8vo. 1811, p. 20."

† "*Dentinum.*—Besides the advantage of a substantive name for an unquestionably distinct tissue under all its modifications in the animal kingdom, the term 'dentine' may be inflected adjectively, and the properties of this tissue be described without the necessity of periphrasis; thus we may speak of the 'dentinal' pulp, 'dentinal' tubes or cells, as distinct from the corresponding properties of the other constituents of a tooth. The term 'dental' will retain its ordinary sense, as relating to the entire tooth or system of teeth."

‡ "*Cæmentum, Cortex osseus*, Tenon. *Crusta petrosa*, Blake."

§ "*Encaustum, Adamas, Substantia vitrea.*"

earthy matter arranged by organic forces in an animal matrix. Here, however, the earth is mainly contained in the canals of the animal membrane, and, in mammals and reptiles, completely fills those canals, which are comparatively wide, whilst their parietes are of extreme tenuity. The hardening salts of the enamel are not only present in far greater proportion than in the other dental tissues, but, in some animals, are peculiarly distinguished by the presence of fluate of lime." P. v.

This passage may be considered as the *argument* or analysis of the whole of the physiology contained in the Introduction, but there are details in this part of the work which demand a more extended notice.

It is certainly to the labours, and we may almost add the genius, of Purkinjé, that we owe the knowledge of all the essential points in the anatomy of the teeth. But it is also very remarkable that, whilst this distinguished observer arrived at all his important conclusions from original researches of his own, ignorant, as we have before stated, that in some of the most considerable, he had been anticipated by Leeuwenhoek—at the same period, or immediately afterwards, certain of his discoveries were also independently made by Retzius, who was equally unacquainted with Purkinjé's labours; and this was not all, for this remarkable race, in which the competitors were really ignorant of each others advances in the course, is completed by our author, who made many observations of the same nature, and exhibited them in his lectures at the College of Surgeons, without being aware that the distinguished men above-named had already anticipated him.

"Thus, the discovery by microscopical examinations that the dentine of the teeth in man and various animals was traversed by minute tubes disposed in a radiated arrangement in lines proceeding every where perpendicularly from the surface of the cavity containing the pulp, may be regarded as established, and to be due principally to the learned and ingenious Purkinjé, who, however, was all the while unconscious that he had been anticipated, as to the main fact, a long time before, by Leeuwenhoek.

"But the tubular structure of ivory is not the only important fact in dental anatomy, made known in the Breslau Thesises of 1835. Purkinjé also discovered that the distinct layer of substance, previously known to surround the fang of the simple teeth of man and many mammalia, contained corpuscles like those which characterize the structure of true bone: and he observed in one instance that this bone-like substance was continued upon the enamel of the crown of a human incisor.

"This fact I have confirmed as regards the human teeth and the simple teeth of many mammals and reptiles. The layer of coronal cement varies in thickness; its tenuity is extreme in the teeth of man and the quadrumana.

"Purkinjé also found that the third substance, *crusta petrosa*, or cement of compound teeth, as those of the horse and ox, was in like manner characterized by the presence of numerous bone-corpuscules or cells; and thus proved that the difference between the so called simple and compound teeth depended, not on the presence of a third and additional substance in the latter, but on its greater abundance and different disposition in the tooth.

"At the time that these observations were being made at Breslau and Berlin, it appears that similar investigations had been set on foot at Stockholm. Professor Retzius of the University in that city informs us that he had been led by the irridescence of the fractured surface of the substance of a tooth to conceive that that appearance was due, as in the crystalline lens, to a fine fibrous structure, and that he communicated his opinions as to the regular arrangement of these fibres to some of his colleagues in 1834; and that the University having obtained, in the summer of 1835, a powerful microscope, by Plessl of Vienna, he commenced a series of more exact researches on the intimate structure of the teeth in man and the lower animals. He operated on thin sections of teeth, both before and after the removal of the earthy matter, by means of acid, and attentively examined the fractured and polished surfaces of the ivory part: he determined the exact arrangement, course, and size of the tubuli in the teeth of different animals, and detected the finer ramifications given off by the tubuli during their divergence,* and the anastomoses of their finest terminal branches with the cells in the intertubular, or as it is sometimes termed interfibrous, tissue.

"Retzius also claims to have discovered the radiated or Purkinjian corpuscles† in the dentine; and to have thus succeeded in displaying a far greater identity between tooth-bone and proper bone than had been before anticipated.

"He exhibited the preparations and drawings illustrative of these interesting observations to Berzelius, Urede, and Professor Wahlberg at the latter end of 1835; being then unacquainted with the discoveries of Purkinjé; and communicated his researches to the Royal Academy of Sciences at Stockholm on the 13th of January, 1836. They were published in the same year in those Transactions and in the following year as a distinct treatise.

"At the early part of that year, 1837, I received from Mr. Darwin many fragments of the teeth of the extinct *Megatherium*, *Megalonyx*, *Mylodon* and *Toxodon*, collected during his travels in South America. Some of these fragments were in a state of incipient decomposition: and my attention was forcibly arrested by the fact that these fragments, instead of being resolved, like the fossil tusks of the mammoth and mastodon, into parallel superimposed conical lamellæ, separated into fine fibres, arranged at right angles to the plane of the layers which, according to the lamellar theory of dental structure, ought to have presented themselves to view. I exhibited the most characteristic of these specimens at my lectures on the teeth, at the Royal College of Surgeons, in May, 1837, and stated that 'the appearances which they presented were inexplicable on the lamellar hypothesis: but that I should investigate the subject further, and endeavour to elucidate the apparent anomaly before the following session.' At the conclusion of that course, I had sections of these fragments prepared for the microscope; and, stimulated by the amount of clearly defined and beautiful structure which they exhibited, I proceeded to examine similar sections of the human teeth and of those of many of the lower animals. The excitement of the research became heightened as the sphere of observation expanded, and I had collected extensive materials for a Treatise expressly on the Structure of Teeth, when the fourth number of Müller's Archiv fur Physiologie, for the year 1837, containing an Analysis of Purkinjé's and Fraenkel's Treatise, came into my hands, in 1837, and awoke me from the dream of discovery in which I had been indulging. I received, shortly after, the fifth number of the same volume of Müller's

---

* "Leeuwenhoek appears to have suspected the existence of such branches; he says, 'upon examining the tubuli round about this small cavity (the pulp-cavity) I perceive that they all arose from thence and spread themselves all round towards the circumference. I endeavoured to examine still farther, beyond the part where this cavity ended, in order to discover whether from these first-formed tubuli others might not arise or branch forth; but this part of nature's work was inscrutable to me.' Hoole's Leeuwenhoek, 4to. p. 113."

† "I have not yet been able to detect the radiated cells or corpuscles in the dentine of the horse's tooth, in which they are described by Retzius; but they are very numerous and conspicuous at the peripheral portion of the dentine of the dugong's grinder, pl. 94."

Archiv, containing Dr. Creplin's German Translation of the Treatise of Retzius, upon the perusal of which I abandoned my intention of publishing those general observations on the structure of the teeth which I had before deemed to be new, but now found to have been mainly anticipated by Purkinjé and Retzius." P. xv.

The essential distinction between what Professor Owen terms "vascular" and "unvascular dentine," appears to us scarcely tenable. The passage from one into the other is almost continuous; at least there are such evident approaches on either side, as to warrant the conclusion that they are mere modifications of the same structure. The procession of the pulp-cavity, with its highly organized contents, in the form of vessels or tubes into the substance of the dentine, is probably the true theory of all the varieties of vascular dentine. The distinction, however, is of great importance in the determination of fossil animals; and here again we have a very valuable element in palæontological science from the researches of Professor Owen.

"The prologation or persistence of cylindrical canals of the pulp-cavity in the dentinal tissue, which is the essential character of vascular dentine, manifests itself under a variety of forms. In mammals and reptiles these canals, which I have termed 'medullary'* from their close analogy with the so called canals of bone, are straight and more or less parallel with each other; they bifurcate, though rarely; and when they anastomose, as in the megatherium, it is by a loop at, or near, the periphery of the vascular dentine. In the teeth of fishes, in which the distinction between the dentinal and osseous tissues is gradually effaced, the medullary canals of the vascular dentine, though in some instances straight and parallel and sparingly divided or united, yet are generally more or less bent, frequently and successively branched, and the subdivisions blended together in so many parts of the tooth as to form a rich reticulation. The calcigerous tubes sent off into the interspaces of the net-work partake of the irregular character of the canals from which they spring, and the meshes fill with a moss-like plexus.

"Closely analogous to this modification of the vascular dentine, but differing in the presence of the radiated cells, is the tissue into which the residue of the pulp is converted in the teeth of certain reptiles, as the Iguanodon, Hylæosaurus and Ichthyosaurus, and of those of a few mammalia, as the Cachalot. This tissue approaches, in the combined presence of medullary canals and calcigerous cells, as closely to that of the skeleton of the species in which it occurs, as the reticulate modification of the vascular dentine in the teeth of fishes does to the osseous tissue of their skeleton. It has been uniformly described by the authors who have observed it, as Cuvier and Conybeare,† as the result of ossification of the pulp.

"If the first described modification of vascular dentine, which forms the chief part of the teeth of the Sloths and Megatherium, be regarded as a fourth dental tissue, this second modification of vascular dentine, from its closer resemblance to bone, might be reckoned as a fifth; in proportion, however, as it resembles bone, so likewise it approaches to the structure of cement." P. xviii.

---

* "This substance was first characterised as a component of tooth, 'distinct from ivory, enamel, cement, and true bone, and as easily recognisable,' in my paper communicated to the British Association, in 1838; loc. cit. p. 137."

† "The teeth in these genera (the Lacretæ) become completely solid, its interior cavity being filled up by the ossification of the pulpy substance."—Trans. Geol. Soc. vol. vi. p. 106.

The substance which, in the so called compound teeth of graminivorous mammals, forms so large and important a constituent of their whole substance, and which, as regards these teeth, is, with much propriety, termed "cement," is found in almost all teeth, and probably may be considered as a necessary element in their structure. Existing, as it does, in the simple teeth only as a thin covering, scarcely perceptible on the surface of the enamel, and encreasing in thickness over the root, the term "cement" is no longer applicable; and we could wish that some word more allusive to its essential situation, exterior to the other parts of the tooth, had been adopted. In its character it nearly resembles bone; and it is by its means that Hunter's well known experiments of uniting the teeth to the comb of a cock, and other curious transplantations, were brought to bear. It is, indeed, the most highly organized part of the teeth, and, as we have ourselves repeatedly verified, it is the seat of exostosis, of union of the roots of contiguous teeth, as well as of several diseased actions, which are not found to take place in any other of the dental constituents.

"This correspondence of the cement, which, when it exists in sufficient quantity, becomes almost identity, with true bone, is illustrated by the varieties of microscopic structure which the cement presents in different classes of animals, and which always correspond with the modifications of the osseous tissue of the skeleton in those animals; thus the cement in the osseous fishes, in which the bone is not characterized by the radiated calcigerous cells, likewise ceases to present that character; and, in reptiles and mammals in which the radiated cells are present in the bone of the skeleton and in the dental cement there is a close conformity as to their size and shape in both tissues.

"The most remarkable modification of mammalian cement is presented by the thick layer of that substance which invests the molars of the extinct megatherium; besides abounding in calcigerous cells it is here traversed by straight, parallel and occasionally bifurcated medullary canals, arranged with regular intervals, and directed from the exterior of the tooth somewhat obliquely to the surface of the unvascular dentine, close to which they anastamose by loops, corresponding with, and opposite to those formed by the medullary canals of the vascular dentine of the same tooth.

"Under every modification the cement is the most highly organized and most vascular of the dental tissues, and its chief use is to form the bond of vital union between the denser and commonly unvascular constituents of the tooth and the bone in which the tooth is implanted. In a few reptiles (now extinct) and in the herbivorous mammalia the cement not only invests the exterior of the teeth, but penetrates their substance in vertical folds, varying in number, form, extent, thickness and degree of complexity, and contributing to maintain that inequality of the grinding surface of the tooth which is essential to its function as an instrument for the comminution of vegetable substances." P. xxi.

Passing over, as we gladly do, the controversial portion of the history of the development of the dentinal structure, we enter upon the theory of our author on the mode in which the three structures of which the teeth consist are developed. It appears that, notwithstanding the approximation which Purkinjé and Schwann made towards the true understanding of these curious processes, yet that much remains to be determined and elucidated on this subject. The main facts which may be considered as established by Purkinjé and Schwann, relative to the formation of dentine, and the changes which the dentinal-pulp undergoes during that process, are the following: the proper tissue of the pulp consists of minute nucleated cells. with capillary vessels and nerves, invested by a dense structureless membrane, which disappears during the formation of the dentine. The superficial pulp-cells assume an elongated form; they correspond in diameter and direction with the tubes of the contiguous cap of dentine. These, or similar cells, are observed in a state of transition into dentine, in the interspace between the pulp, and the previously formed cap of dentine; they adhere to the latter when displaced from the pulp.

"The chief points that remain to be determined are the relation of the dentinal pulp to the transitional cells, between it and the dentine; the nature of the transition, and the relation of the cells to the dentinal tubes, and the intertubular tissue." P. xxxvii.

Such then was the state of our information on the formation of dentine, when Professor Owen took the matter up thoroughly to investigate it. Before, however, we proceed to give at some length, and in our author's own words, his account of the interesting process in question, it is but fair to state that the primâ-facia condition of the parts standing in relation to these organs, had given rise to a theory of their development, to which all previous authors had leaned, and from a tendency to which it was difficult to emancipate the mind. The "excretion theory" was doubtless that of Hunter, and was supported by Blake, and more distinctly by Bell, who considers the "proper membrane of the pulp" as the secreting surface, the pulp being merely the mould on which the ivory is successively deposited. If the membrane alluded to be identical with the pre-formative membrane of Raschkow, as stated by Professor Owen, then it is certain that its office, as assigned by Bell, must be altogether assumed, or that the description of Raschkow of its existence *exterior* to the dentine substance must be erroneous. We believe, however, that further observations still are required, with regard to the office, the structure, and even the existence, of this membrane.

In now giving a full account of Professor Owen's investigations, which we do in his own words, because we find it very difficult to convey, in a condensed form, any adequate idea of their results,—we offer no apology for the length of our quotations, nor, on the other hand, for the omission of the lengthy, and not very agreeable, controversial notes, which, however they may be considered necessary for the purpose of settling an obstinate controversy, certainly do not contribute to the clear understanding of the author's views, nor to the interest or pleasure of the reader.

"The following are the progressive steps of the calcifying processes, according to my microscopic researches on the formation of the different substances which compose the more complex teeth of Reptiles and Mammals, pursued in various species of both classes, but chiefly in the higher organized domestic animals.

"Three formative organs are developed, as already described, for the three principal or normal dental tissues. The dentinal pulp, or pulp proper, for the dentine; the 'capsule' for the cement, and the 'enamel-pulp' for the enamel. The essential fundamental structure of each formative organ is cellular; but the cells differ in each organ, and derive their specific characters from the properties and metamorphoses of their nucleus, upon which the specific microscopical characters of the resulting calcified substances depend.

"In the cells of the dentinal pulp the nucleus fills the parent cell with a progeny of nucleoli before the work of calcification begins: in the enamel-pulp the nucleus of the cell disappears, like the cytoblast of the embryo plant in the formation of most vegetable tissues: in the cells of the capsule, the nucleus neither perishes nor propagates, but retains its individuality, and gives origin to the most characteristic feature of the cement, viz:—the radiated cell.

"The primordial material of each constituent of the tooth-matrix is derived from the blood, and special arrangements of the blood-vessels pre-exist to the development and growth of the constituent substances. A pencil of capillaries is directed to a particular spot in the primitive dentiparous groove, and terminates there by a looped net-work, from which spot a group of nucleated cells begins to arise in the form of a papilla." P. xlii.

"The primary dentinal papilla and its capsule rapidly increase by successive additions of nucleated cells, apparently derived from material supplied by the capillary plexus at the base; the capillaries now begin to penetrate the substance of the pulp itself, where they present a sub-parallel or slightly diverging penicillate arrangement, but preserve their looped and reticulate termination near the apex of the pulp. Fine branches of nerves accompany the capillaries, and terminate also in loops." P. xliii.

"The primary cells and the capillary vessels and nerves are imbedded in, and supported by a homogeneous, minutely sub-granular, mucilaginous substance, the 'blastema.' The cells which are smallest at the base of the pulp, and have large, simple, subgranular nuclei soon fall into linear series directed towards the periphery of the pulp: where the cells are in close proximity with that periphery, they become more closely aggregated, increase in size, and present the following changes in their interior. A pellucid point appears in the centre of the nucleus which increases in size and becomes more opake around that central point, rendering the compressorium requisite for its demonstration. A division of the nucleus in the course of its long axis is next observed. In the larger and more elongated cells, still nearer the periphery of the pulp, a subdivision of the nuclei has taken place, and the subdivisions become elongated with their long axes vertical, or nearly so, to the plane of the pulp, and to the field of calcification. The subdivided and elongated nuclei become attached by their extremities to the corresponding nuclei of the cells in advance; and the attached extremities become confluent. Whilst these changes are proceeding, the calcareous salts of the surrounding plasma begin to be accumulated in the interior of the cells, and to be aggregated in a semi-transparent state around the central granular part of the elongated nuclei, which now present the character of secondary cells, and the salts occupy, in a still clearer and more compact state, the interspaces of such cells: the elongated granular matter of the terminally confluent secondary cells establishes the area of the tubes, by resisting, as it would seem, the encroachment of the calcareous salts; the nuclear tracts receiving a smaller proportion of the salts, in the condition of minute disgregated particles, which are usually arranged in a linear series of nodules, and contribute to cause the white colour of the moniliform area of the tube when viewed by reflected light, and its opacity when viewed by transmitted light. Thus the primitive existence of the granular nuclei, their multiplication in the primary or parent cell, their elongated form, their serial arrangement end to end, and terminal confluence, are indicated in the calcified pulp by the areæ of the dentinal tubes; the interspaces of the metamorphosed nuclei being occupied by calcareous salts in a clearer and more compact state, with evidence, however, of a distinctness of the nucleolar membrane or secondary cell from the cavity of the common containing cell, which sustains the interpretation of the proper parietes of the dentinal tube. The indications of the primitive boundary or proper parietes of the parent-cell are in like manner more or less distinctly retained, through a modification of the arrangement of the calcareous salts in the boundaries and in the interspaces of the cells. The salts are sometimes blended with the blastema in these interspaces in a disgregated condition which renders them almost as opake as the areæ of the tubes." P. xliv.

We reget that want of room prevents our extracting the remainder of the elaborate details of the formation of this principal constituent of the teeth, which will not, however, admit of condensation. The formation of the enamel is not less minutely described, but the following extract will suffice to shew the general views entertained by the author.

"The enamel pulp differs from the dentinal pulp at its first formation by the more fluid state of its blastema and by the fewer and more minute cells which it contains. The source of this fluid blastema appears to be the free inner vascular surface of the capsule. As it approaches the dentinal pulp the blastema acquires more consistence by an increased number of its granules, and it contains more numerous and larger cells; many of these show a nuclear spot: others a nucleus and nucleolus: the spherical nucleolar cells in the part of the blastema further from the capsule are so numerous as to form an aggregate mass, with a small quantity of the condensed blastema in the minute interspaces left between the cells, which are pressed together into hexagonal or polygonal forms. In this state they constitute a great part of the enamel pulp, which is of considerable extent in the complex molar teeth of the Ruminants. The appearance produced by these aggregated cells, in a section of the tooth-matrix of a calf's molar, is compared by Raschkow to the actinenchyma of certain vegetable tissues, and the connecting condensed blastema to threads of cellular tissue. The field of the final metamorphosis of the cells into the moulds for the reception of the solidifying salts is confined to close contiguity with the surface of the dentinal pulp.

"Here the cells increase in length, lose all trace of their nucleus, and become converted into long and slender cylinders usually pointed at both ends, and pressed by mutual contact into a prismatic form. These cylinders have the property of imbibing the calcareous salts of the enamel from the plasmatic fluid, and of compacting them in a clear and almost crystalline state in the interior: the disappearance of the nucleus being evidently the condition of the absence of any permanent cavity, cell, canal, or other modification of the mineral matter, at least in the enamel fibres of the calf. In the human subject it is probable that the cavity of the cylinder may be subdivided by a multiplication of delicate nucleoli into compartments; or that the remains of such multiplied nucleoli may cause a modification of the walls of the cylinder, and so produce the characteristic transverse striæ of the enamel-fibre. This appearance is not present in the enamel of the frog's tooth, nor in that of the teeth of the hog, or calf, in which animals my observations of the development of this tissue have been chiefly made. As the development proceeds, the cells in immediate contiguity with the calcified prisms undergo the same changes as their predecessors, and become united to them by their peripheral pointed extremities, whilst the fluid plasmatic contents of the cells are exchanged for the dense salts of which the enamel is chiefly composed. The selective surface formed by the organic membrane of the cell would seem to be destroyed by the very pressure resulting from its own action, and exerted by the contents of the closely-packed contiguous prisms, when the cavities of the cells are completely filled. The membrane ceases at least to be distinguishable under the microscope, from the solid contents of the cell, except at that surface of the enamel next the capsule, and which is still in progress of growth." P. lix.

The remaining structure of which the development is to be described is the "cement," which is produced by the sac or capsule, the most external of the rudiments or formative structures of these organs.

"The blastema or fundamental tissue of the capsule is, at first, semitransparent and of a pearly or opaline colour; but is soon richly ornamented by the plexiform distribution of the blood-vessels. As the period of its calcification approaches, which is later than that of the dentinal pulp, it becomes denser, and exhibits numerous nucleated cells. The blastema itself presents more evidently a fine cellular or granular structure in which the calcareous salts are impacted in a comparatively clear state constituting the framework of the cemental tissue. The characteristic features of this tissue are due to the action of the proper nucleated cells upon the salts of the plasma diffused through the blastema in which those cells are imbeded. The cells being characterised by a single large granular nucleus which almost fills the clear area of the cell itself. If, when the formation of the cement has begun in the incisor or molar of a colt, one of the detached specs of that substance, with the surrounding and adhering part of the inner surface of the capsule in which it is imbedded, be examined, these nucleated cells are seen, closely aggregated around the calcified part, in concentric rows; the cells of which are further apart as the rows recede from the field of calcification. Those next the cement rest in cup-shaped cavities in the periphery of the calcified part just as the first calcified cells of the thick cement which covers the crown of a complex molar are lodged in cavities on the exterior of the enamel. These exterior cavities of the cement are formed by centrifugal extension of the calcifying process in the blastema in which the cells are imbedded. The calcareous salts penetrate in a clearer and more compact state the cavity of the cell, but their progress is arrested apparently by the nucleus which maintains an irregular area, partly occupied by the salts in a subgranular opake condition, but chiefly concerned in the reception and transit of the plasmatic fluid which enters and escapes by the minute tubes which are subsequently developed from the nucleolar cavity as calcification proceeds. The radiated cells or cavities thus formed, are the most common characteristic of the cement, but not the constant one. The layer of the capsule which surrounds the crown of the human teeth and of the simple teeth of quadrumana and carnivora, consists simply of the granular blastema, without nucleated cells, and the radiated corpuscles are, consequently, not developed in the cement which results from its calcification. In the thicker parts of the inflected folds of the capsule of the complex teeth of the herbivora traces of the vascularity of that part of the matrix are persistent, the blastema calcifying around certain of the capillaries and forming the medullary canals. The varieties of these canals are traversed by minute tubules continued from or communicating with the radiated cells. These tubules, and the more parallel ones which traverse the thickness of the cement in many mammalia, are the remains of linear series of the minute granules of the blastema.

"In the deep sockets of the teeth of persistent growth the matrix is maintained by the constant additions of new blastema and cell-material to the bases of the dentinal, enamel and cemental pulps." P. lxi.

It is worthy of particular observation here, that, on reverting to the works of former authors on the anatomy of the teeth, of those whose labours were antecedent to the period of the revival of microscopy, the Hunters and Blakes and their followers, we find all the above-mentioned formative structures described with more or less accuracy, and assigning to each its true and appropriate office. It is no new discovery that the pulp forms the matrix of the dentine, that the enamel owes its origin to one portion of the capsule and the cement to another. The dentinal pulp is described by Hunter as "a pulpy substance which is pretty firm in its texture, transparent excepting at the surface where it adheres to the jaw." "These pulpy substances are very vascular."* The beginning of ossification is by one point or more according to the kind of tooth. * * The

* Hunter on the Teeth, Bell's Ed. p. 38.

ossifications in their progress become thicker and thicker where they first began, but encrease faster on the edges of the teeth. * * * It gradually surrounds the pulp, till the whole is covered by bone, except the under surface; and while the ossifications advance, that part of the pulp which is covered by bone *is always more vascular than the part which is not yet covered.* The adhesion of the pulp to the new-formed tooth or bone is very slight, for it can always be separated from it *without any apparent violence,* nor are there any vessels going from the one to the other."

Blake gives nearly a parallel description of the structures in question. He considers, however, that the pulp is derived from "the vessels which enter the lower part of the sacs" or capsules. He states that the pulp is very vascular; and adds that, "as the bone of the tooth increases in thickness, the pulp is proportionably diminished, and seems, as it were, converted into bone;" but he goes on to disprove this by observing that "its connexion with the bony part is very slight, except at its extreme elastic edge. * * * When the shell is removed, the pulp appears covered with a very delicate membrane, on which the vessels form a net-work."* This is clearly the "proper membrane of the pulp, subsequently described by Bell as the secreting organ of the ivory, and believed by Owen to be identical with the "preformative membrane" of Raschkow.

The opinions promulgated by the author just named on this subject do not differ materially from those of his predecessors. His theory however is, that the membrane just named, the "proper membrane of the pulp," is the true secreting organ of the ivory;† and in another place, in his edition of Hunter, he more fully declares that the pulp "constitutes only the mould upon which the ossification is formed, between which and the pulp is placed a membrane of extreme tenuity, * * * slightly attached to the surface of the pulp, which it completely covers; and it is from the outer surface of this membrane that the bone (ivory) is secreted."‡

* Blake, "Essay on the Structure and Formation of the Teeth," &c., p, 5, *et seq.*

† Bell on the Teeth," p. 54.

‡ Bell in Hunter on the Teeth, p. 38.

|| Hunter. *L. c.*, p. 41.

the capsule and the enamel pulp, are concerned in the production of the enamel in man, because it "seems evident" that they are in granivorous quadrupeds. The mode in which the final crystalline form of this substance is accounted for is worthy of notice. "It is a calcareous earth, thrown out from these parts, which act here as a gland. After it is secreted, the earth is attracted by the bony part of the tooth which is already formed, and upon that surface it crystallizes. The operation is similar to the formation of the shell of the egg, of the stone in the kidneys or bladder, and the gall-stone." Of the formation of cement Hunter gives no account.

Blake was the first author by whom the true relation of the cement to the tooth in the graminivorous quadrupeds was understood, but he appears somewhat confused in his description of the mode of its production. Of the enamel, or, as he terms it, "*cortex striatus*," he states that it is deposited by the inner surface of the *sac* or capsule. "The sacs or membranes which surround the cells * * * can easily be separated into two lamellæ, the external of which is spongy and full of vessels. The internal one is more tender and delicate, and seems to contain no vessels capable of conveying red blood." Hunter, on the other hand, declares that the external layer is "soft and spongy, without any vessels; the other is much firmer, and extremely vascular." Fox declares, and Bell agrees with him, that both are vascular! The account which Blake gives of the office of the capsule is so near the truth, and so anticipatory of the present theories, that, in justice to him, we think it right to give it in his own words. "It appears that the internal part of the membranes [enamel-pulp ?] secretes the earthy matter of the *cortex striatus* [enamel], and that, as soon as it has performed its function, it is wasted or destroyed; for its external lamella, as soon as the upper part of the *cortex striatus* is crystallised, begins to deposit on its surface a substance differing from either the bony part or the cortex striatus, being harder and more brittle than the former, but less so than the latter."* It appears to us that this statement comes as near the truth as it was possible, for one who had not the advantage of microscopical examination to aid him.

There are a few remarkable discrepancies which cannot but strike us on comparing the statements of the older authors whom we have just quoted with the theories enunciated by the supporters of the doctrine of the *conversion* of the dentinal pulp into dentine, and of the enamel pulp into enamel. The first and most obvious is the asserted existence of an enduring vascular membrane covering the surface of the pulp, and interposed between it and the progressing dentine. The pulp is declared by Blake to be covered with a very delicate membrane, on which the vessels form a net-work," and even goes so far as to consider it as "a propagation of the periosteum of the jaw." Bell, as we have seen, is still more particular on this point. Connected immediately with this assertion, is the statement made by all three of the authors above quoted, that the shell of ivory is so easily detached from the surface of the pulp, as scarcely to offer any perceptible resistance. Hunter's assertion, that there are no

---

* Blake. *L. c.*, p. 84.

vessels going from the pulp to the shell of bone, must be considered as another mode of expressing this entire absence of any obvious connexion between the two parts. Now, if these assertions be true, we confess we are at a loss how to reconcile them to the gradual *conversion* of the substance of the pulp into the substance of the dentine, especially considering that the whole shell of dentine is hard, and the whole substance of the pulp still soft;—there appears to be no equal and continuous transition between the soft gelatinous texture of the pulp, and the solidity of the dentinal shell or cap. Again—the very distinct lamellar fracture or concentric separation of layers of dentine, produced by partial calcination, and still more remarkably by long inhumation, as in the case of fossil elephants' tusks, deserves much consideration when associated with the other facts above-mentioned. We do not make these remarks as throwing any doubt on the statements of Professor Owen, or of the other distinguished men who support a similar theory to his, but only in order to call attention to these discrepancies, and to express our feeling that they have been somewhat too slightly passed over in the present work.

We have thus endeavoured to give as complete a view of the introductory portion of this work as our limits would admit—and we have now to offer a sketch of the general arrangement of the principal objects of the book, and of the conclusions which are founded upon the mass of important details contained in it.

The general arrangement of the body of the work is as follows. Commencing with the fishes, we have the principal groups of "Cyclostomes," "Plagiostomes," "Gonoid fishes," "Ctenoid fishes," and "Cycloid fishes," with their different subordinate groups. The Batrachians are not treated as a distinct class from the Reptiles, but only as one of the primary groups of the class, and, including the interesting genus of *Labyrinthodon*, are exhibited under thirteen different forms: the Ophidians and the Saurians follow, the Enaliosaurians being included in the latter. The arrangement of the Mammals appears to be nearly arbitrary. After some observations on the horny teeth of the monotremes, the blubber whales, &c. the remainder are classed in the following series: Bruta, Cetacea, Marsupialia, Rodentia, Insectivora, Cheiroptera, Quadrumana, Bimana, Carnivora, and Ungulata, including "Isodactyle ungulates," "Anisodactyle ungulates," and "Proboscidians." The teeth in each of the principal groups are treated in full and admirable detail under the several heads of number, form, situation, attachment, substance, chemical composition, and development.

It would perhaps border upon hypercriticism to animadvert upon the inconsistencies of nomenclature, which are very obvious and very frequent. It may be answered that a work like this, the object of which is purely anatomical and physiological, needs not the same attention to accuracy and consistency in the subordinate matter of terminology, as if it belonged to a subject of systematic and formal zoology. We suppose the defence, and willingly accept it; and proceed to consider briefly the generalities of each principal division or group of animals.

It is of course with the full conviction of the truth of the "conversion theory" that the whole of the details of structure and development throughout the book are stated and followed out; and whatever may be the difficulties which meet us on a more superficial view, and to which we have already adverted, the entire consonance of all our author's facts with this theory, and the impossibility of rendering many of those facts compatible with the "excretion theory," will, we doubt not, tend to establish the former firmly in the minds of all the attentive students of this book, until future observations may tend to modify, or (we hesitate to suppose such a thing even possible) to subvert it.

We pass over the elaborate and interesting details of the form and situation of the teeth in the various tribes of fishes, as they are matters which may be found more or less satisfactorily described in other places. We would merely observe that the *final cause* is, in all instances, admirably made out. A brief example, however, which meets us at the very commencement, we cannot help extracting, referring, at the same time, to the beautiful plate (46) which illustrates it.

"If the engineer would study the model of a dome of unusual strength, and so supported as to relieve from its pressure the floor of a vaulted chamber beneath, let him make a vertical section of one of the crushing pharyngeal teeth of the wrasse. The base of this tooth is slightly contracted, and is implanted in a shallow, circular cavity, the rounded margin of which is adapted to a circular groove in the contracted part of the base; the margin of the tooth, which immediately transmits the pressure to the bone, is strengthened by an inwardly projecting convex ridge. The masonry of this internal buttress and of the dome itself, is composed of hollow columns, every one of which is placed so as best to resist or transmit in the due direction, the superincumbent pressure." P. 9.

The substance of the teeth in certain fishes is more complex and more variable than in any other class.

"The greater number of fishes have their teeth composed of an osseous substance, somewhat denser than the jaws to which they are affixed. In some instances, as in the teeth of the flying-fish (*Exocœtus*), and sucking-fish (*Remora*), the substance of the tooth is uniform, and not covered by a layer of a denser texture. In others, as the shark, sphyræna, &c., the tooth is coated with a dense, shining, enamel-like substance; but this is not true enamel, nor the product of a distinct organ; it differs from the body of the tooth only in the greater proportion of the earthy particles, their more minute diffusion through the gelatinous basis, and the more parallel arrangement of the calcigerous tubes; but it is developed in and by the same matrix, and, resulting from the calcification of its external layer, is the first part of the tooth which is formed. In the *Sargus* and *Balistes*, the dentine, or proper osseous substance of the tooth, is harder than that of the fishes last cited; and is covered with a thick layer of a denser substance, developed by a distinct organ, and differing from the enamel of the higher animals only in the more complicated and organized mode of deposition of the earthy particles. The ossification of the capsule of the matrix gives the enamel of the teeth of the file-fish, and some others, a thin coating of a third substance analogous to the 'cæmentum, or crusta petrosa,' of the mammalian teeth. And in the pharyngeal teeth of the parrot-fish, a fourth substance is added to the structure of the tooth by the coarser ossification of the pulp, after its peripheral portion has been converted into the dense ivory. The teeth, thus consisting of dentine, enamel, cement, and coarse bone, are the most complicated as regards their substance that have yet been discovered." P. 9.

With regard to the intimate structure of the teeth in this class, the author states that, there are

"At least four principal modifications of the tubular structure of the teeth of fishes. Premising that the essential character of this structure is a *cavitas pulpi*, or medullary canal, from which the calcigerous tubes radiate, the first modification which may be noticed is where the tooth is traversed by a number of equidistant and parallel medullary canals, each canal and its system of medullary tubes representing a cylindrical or prismatic denticle, and being separated from the contiguous denticles by a thin coat of bone or cement. This modification is exemplified in the rostral teeth of the saw-fish (*Pristis*), the tessellated teeth of the eagle-rays (*Myliobates, Zygobates, &c.*) and the maxillary plates of the chimæroids. The dense dental case of the jaws of the parrot-fishes (*Scarus*), may likewise be regarded as an extreme instance of this modification, and we shall find the same structure reappearing in some of the inferior genera of the mammiferous class. In the parrot-fishes, the denticles are quite distinct from one another, but in the saw-fish, chimæra, and eagle-rays, the contiguous medullary canals occasionally anastomose together. In the chimæroid fishes these anastomoses are more numerous, and the boundaries of the component denticles less distinct, so that they form a transition between the preceding, and what may be regarded as the second variety of the tubular structure.

"In this modification the substance of the tooth is traversed by medullary canals, somewhat less regularly equidistant and less parallel than in the first; having the boundaries of their respective systems of radiated calcigerous tubes indicated by the minute calcigerous cells, with which the terminal branches of those tubes communicate; these boundaries being more or less obscured by the terminal branches of the calcigerous tubes extending across into the interspaces of the corresponding branches of an adjoining system of tubes, and anastomosing with them immediately, or through intervening dilatations or cells. The medullary canals here dichotomize more frequently than in the first modification; their anastomoses are more numerous, and the whole tooth, which is generally of large size, is consequently more individualized and compacted. The teeth of the Port-Jackson shark (*Cestracion Phillippi,*) afford a good example of this modification, which also prevails in those of the extinct genera *Ptychodus, Psammodus, Helcdus, Ctenoptychius, &c.* In the teeth of the extinct *Acrodus*, the medullary canals, which likewise traverse in great numbers the body of the tooth, assume a more or less wavy course; and this disposition, combined with their numerous anastomoses, leads to the third modification, which at the same time is the most common and characteristic of the dental structure, in the class of fishes.

"In teeth manifesting this variety of tubular structure, the dentine is permeated by a network of medullary canals, of which the interspaces are occupied by the calciger ous tubes and cells. The medullary canals are directly continued from those of the common bone with which the base of the tooth is anchylosed, or into which it has been converted. As the medullary canals proceed through the tooth, they maintain a course more or less parallel, and more or less straight or wavy; but they ramify abundantly, and gradually diminish in calibre as they approach the surface of the tooth. The illustrations of this modification of the dental structure in the present work, are taken from the teeth of the extinct *Lamna, Dictyodus*, and *Saurocephalus*, and from those of the recent *Sphyræna* and *Acanthurus.* In the latter genus the dendritic arrangement of the medullary tubes recognized by Mr. André, has subsequently been figured by V. Born. V. Born and Retzius have described a similar structure in the teeth of the wolf-fish (*Anarrhichas*), of which Mr. Nasmyth has given a figure in his useful translation of some of the recent treatises on dental anatomy.

"The reticulate medullary tubes pervade the structure of the teeth of the percoid, sciænoid, cottoid, and gobioid families of fishes; and of those of the *Capros, Naseus*, and other genera of the *Theuties* of Cuvier, besides the teeth of the *Acanthuri* already cited. A similar reticulate structure is common to the teeth of the *Chætodontes* and the *Pleuronectes:* in the cycloid fishes, we find it almost universal in the scomberoid, lucioid, salmonoid, and clupeoid families: it is exchanged for a higher type of structure in the maxillary teeth of the lophioid fishes, and in the pharyngeal teeth of the cyprinoids, but it again reappears in the teeth of the blennioid, gadoid, and muræenoid families; and the same coarse bone-like structure pervades the dental plates of the supposed amphibious *Lepidosiren*.

"The higher type of structure just alluded to is that which characterises the teeth of most reptiles and mammalia. Here the dentine consists of a single medullary or pulp canal, and a single system of calcigerous tubes radiating from the central or sub-central canal, at right angles to the periphery of the tooth. The teeth of the extinct sauroid fishes and pycnodonts, the maxillary teeth of the existing file-fishes (*Balistes*), and angler (*Lophius*), the incisors, canines, and molars of the breams or sparoid fishes, the pharyngeal pavement-teeth of the wrasse-tribe (*Labridæ*), the maxillary and pharyngeal denticles of the scari, and the lamelliform denticles of the crop-fishes, *diodon* and *tetrodon*, likewise the maxillary teeth of some of the genera of sharks and rays, afford examples of this structure." P. 13.

The following remarks on the changes from one of these modifications of dental structure to another are interesting and important, as exhibiting the conclusion to which a philosophical mind has been brought by an immense accumulation of facts, acutely reasoned upon, respecting the long debated question of the true organization and life of these organs; and they refute in a few words the hypothetical application of such terms as "la partie morte," unorganised structures, &c. which have been so often assigned to the solid substance of the teeth.

"The uniform result of my researches, on the structure of the teeth in all grades of vertebrate animals, and in their natural and diseased states, has been a conviction of the untruthfulness of the terms inert, dead, and unorganised as applied to the substance of any tooth whatever. Extra-vascular undoubtedly is all that portion which consists of the calcigerous tubes; the capillary circulation is confined to the pulp or medullary canals; but since every secretive process and the development of the primordial cells of every tissue are due to changes produced in the liquor sanguinis transuded from and beyond the sphere of the ultimate capillaries, the absence of these vessels in the dense dental substance is as little conclusive against its vital and organized nature, as it would be to prove the inert condition of the germinal membrane of the ovum before the thirtieth hour of incubation." P. 13.

Mr. Owen's theory of the development of the teeth in fishes, is in accordance with that of the osseous structures generally; that is to say, that although they are formed according to the general laws of dental development, "the process in many instances does not extend beyond the earlier and simpler stages observable in the higher classes of animals. In all fishes, as in other vertebrate animals, the first step is the production of a simple papilla from the free surface of either the soft external integument, as in the young *Pristis*,* or of the mucous membrane of the mouth, as in the rest of the class. In these primitive papillæ there can be very early

* "A very close analogy exists between the dermal bony tubercles and spines of the cartilaginous fishes and their teeth. The thick enamelled scales of the ganoid fishes of Agassiz exhibit an organization similar to that of the teeth: the system of minute parallel tubes, with their branches and anastomoses, in the thick scales of the extinct *lepidotus*, is as complicated as in many teeth, and equally militates against the theory of formation by transudation of layers being applied, at least, to the ganoid scales."

distinguished a cavity containing fluid, and a dense membrane, (*membrana propria pulpi*) surrounding the cavity, and itself covered by the thin external buccal mucous membrane, which gradually becomes more and more attenuated as the papilla increases in size. In some fishes, as the sharks and rays, the dental papillæ do not sink into the substance of the vascular membrane from which they grow, but become buried in depressions of an opposite fold of the same membrane; these depressions enlarging with the growth of the papillæ, and forming the cavities or capsules in which the development of the tooth is completed. They differ from the capsules of the matrix of the mammiferous tooth in having no organic connexion with the pulp, and no attachment to its base: the teeth when fully formed are gradually withdrawn from the above described extraneous capsules, to take their place and assume the erect position on the alveolar border of the jaws.

"Here, therefore, is represented on a large and, as it were, persistent scale, the first and transitory papillary stage of the development of the mammalian teeth; and the simple crescentic cartilaginous maxillary plate, with the mucous groove behind it containing the germinal papillæ of the teeth, offers in the shark a magnified representation of the earliest condition of the jaws and teeth in the human embryo." In other cases, however, as in the Balistes, Sparoids, Sphyræna, Scarus, &c. the teeth go through the entire process of formation which is observed in the mammals.

There is a remarkable observation of Professor Owen on this subject, which if correct, would at once decide the question before alluded to, of the office and duration of the "proper membrane of the pulp," to which some observers have assigned the office of *secreting* the dentine. "In the shark, and all those fishes in which the teeth are completely formed without going beyond the papillary stage of development, there is no distinct enamel-pulp; the dense exterior layer of the tooth is formed by the calcification of the 'membrana propria' of the pulp, which therefore precedes the formation of the ordinary dentine."

But our space reminds us that we must quit the present class, and pass on to the Reptiles.

The importance of the dental apparatus in determining the affinities of extinct Saurian and other reptiles, has long been known. The external characters of these enduring remnants of an ancient creation have been, with more or less correctness, employed as means of distinguishing or of associating animals of this class, in relation to those of our own period. But until the present work appeared, or at least until it's author made known the results of his observations, the subject has been very imperfectly understood, and the full import of these characters only in a slight degree developed. The intimate structure of the dentine has, however, now become as it were a test of these relations; and in future, a slice only of a tooth, examined with the eye of a practised observer, and one fully acquainted with the minute structural character on which the distinctions or relations depend, will be sufficient to indicate the place of an animal of which no other remains may have come down to us.

It is unnecessary for us to enter into any detail of the external characters and the relations of the teeth to the parts with which they are connected. These points are stated in the general observations on the class.

There is, however, one remark, with reference to the attachment of the teeth in reptilia, which bears upon an important physiological question. It is that the modifications of the mode of attachment of the teeth of these animals offer a close analogy to some of the transitory conditions of the human teeth.

"There is a period, for example,* when the primititve dental papillæ are not defended by either an outer or an inner alveolar process, any more than their gigantic calcified analogues in the extinct Mosasaur. There is another stage† in which the groove containing the dental germs is defended by a single external cartilaginous alveolar ridge: this condition is permanently typified in most existing lizards. Next there is developed an internal alveolar plate, and the sacs and pulps of the teeth sink into a deep but continuous groove, in which traces of transverse partitions soon make their appearance: in the ancient Ichthyosaur the relation of the jaws to the teeth never advanced beyond this stage. Finally, the dental groove is divided by complete partitions,‡ and a separate socket is formed for each tooth, and this stage of development is attained in the highest organized reptiles, as in the crocodile." P. 183.

The following observations on the structure of the teeth in this class, are highly interesting, although it is impossible to convey any correct idea of the beauty and complication of the structures described in the absence of the engravings in which they are exhibited.

"The varieties of dental structure are few in the reptiles as compared with either fishes or mammals, and its most complicated condition arises from the interblending of the dentinal and other substances rather than from modifications of the tissues themselves. In the teeth of most reptiles the intimate structure of the dentine corresponds with that which has been described as its fourth type or modification in the teeth of fishes, and which is the prevailing structure of mammalian dentine, viz: the radiation of a system of minute calcigerous tubes from a single pulp-cavity, at right angles to the external surface of the tooth. The most essential modification of this structure is the intermingling of cylindrical processes of the pulp cavity, in the form of medullary canals, with the finer tubular structure. Another modification is that in which the dentine maintains its normal structure, but is folded inwardly upon itself, so as to produce a deep longitudinal indentation on one side of the tooth: it is the expansion of the bottom of such a longitudinal deep fold that forms the central canal of the venom-fang of the serpent; but a glance at Pl. 65 A, will show that, notwithstanding the singularly modified disposition of the dentine, its structure remains unaltered: and although the pulp-cavity is reduced to the form of a crescentic fissure, the calcigerous tubes continue to radiate from it, according to the usual law. By a similar inflection of many vertical longitudinal folds of the external cement and external surface of the tooth, at regular intervals around the entire circumference of the tooth, and by a corresponding extension of radiated processes of the pulp-cavity and dentine into the interspaces of such inflected and converging folds, a modification of dental structure is established in certain extinct reptiles, which, by the various sinuosities of the interblended folds of cement and processes of dentine, with the partial dilatations of the radiated pulp-cavity, produces the most complicated structure that has yet been met with in

* "At the sixth month, see Mr. Goodsir, On the development of the Human Teeth—Edinburgh Medical and Surgical Journal, No. 138."

† "At the seventh or eighth week.—Ibid."

‡ "At the sixth month.—Ibid."

the teeth of any animal. But this complication is nevertheless referable to a modification of form or arrangement rather than of structure of the dental tissues: the calcigerous tubes in each sinuous lobe of dentine, in the most complex tooth of the Labyrinthodon, exhibit the same general disposition and course as in the fang of the Serpent, and in the still more simple tooth of the Saurian.

"In the Iguanodon the fine-tubed dentine is traversed by medullary canals which run at pretty definite intervals through the dentine, parallel with the calcigerous tubes, as in that coarser kind of dentine which characterizes the teeth of the sloth and the megatherium, and which in connection with the complex form of the teeth of the Iguanodon, peculiarly adapted that gigantic reptile for a vegetable diet.

"The cement is simply and minutely cellular upon the crown of the tooth, but it exhibits the radiated cells at the base of the tooth in the anourous Batrachians, and Saurians. The enamel is subtransparent, dense, and minutely fibrous in all the reptiles which have their teeth defended by this substance." P. 185.

The law of the arrestation of Development in an organ, in different stages of its progress, according to the advance of the different groups of animals towards the highest type, has already been shewn with relation to the teeth, in those of many fishes, in which these organs do not proceed beyond the first or papillary stage. It is further illustrated in the present class, by an advance in their organization to the next stage.

"The teeth of reptiles are never completed, as in certain fishes, at the first or papillary stage; but the pulp sinks into a follicle, and becomes inclosed by a capsule: the process of development, however, never offers the eruptive stage, in the sense in which this is usually understood, as signifying the extrication of the young tooth from a closed alveolus.

"The completion of a tooth is soon followed by preparation for its removal and succession: the faculty of developing new tooth-germs seems to be unlimited in the present class, and the phenomena of dental decadence and replacement are manifested at every period of life: the number of teeth is generally the same in each successive series, and the difference of size presented by the teeth of different and distant series is considerable.

"The new germ is always developed, in the first instance, at the side of the base of the old tooth, never in the cavity of the base; the crocodiles form no exception to this rule. The poison-fangs of serpents succeed each other from behind forwards; in almost every other instance, the germ of the successional tooth is developed at the inner side of the base of its predecessor. In the frog the dental germ makes its appearance in the form of a papilla developed from the bottom and towards the outer side of a small fissure in the mucous membrane or gum that fills up the shallow groove at the inner side of the alveolar parapet and its adherent teeth; the papilla is soon enveloped by a capsular process of the surrounding membrane: there is a small enamel pulp developed from the capsule opposite the apex of the tooth; the deposition of the earthy salts in this mould is accompanied by ossification of the capsule, which afterwards proceeds *pari passu* with the calcification of the dentinal papilla or pulp: so that, with the exception of its base, the surface of the uncalcified part of the pulp alone remains normally unadherent to the capsule.

"As the tooth acquires hardness and size it presses against the base of the contiguous attached tooth, causes a progressive absorption of that part, and finally undermines, displaces and replaces its predecessor. The number of nascent matrices of the successional teeth is so great in the frog, and they are crowded so close together, that it is not unusual to find the capsules of contiguous tooth-germs becoming adherent together as their ossification proceeds. After a brief maceration the soft gum may be stripped from the shallow alveolar depression and the younger tooth-germs in different stages of growth are brought away with it.

"The mode of development of the teeth of serpents does not differ essentially from that of the teeth of the Batrachian above described, except in the relation of the papillæ of the successional poison-fangs to the branch of the poison duct that traverses the cavity of the loose mucous gum in which they are developed." P. 186.

Compelled as we are by want of space to limit our extracts and analysis to the generalities of each class, we resist the temptation, no easy task, to transfer to our pages the account of the Labyrinthodon, which is not only interesting as affording an additional example of the value of the characters of dentinal structure in reference to the relations of animals, but also as exhibiting the most strikingly beautiful structure perhaps to be found in the whole range of these exhaustless varieties. We pass therefore to the concluding class, the Mammals.

"The teeth of the Mammalia usually consist of hard or unvascular dentine, defended at the crown with an investment of enamel, and everywhere surrounded by a coat of cement. The coronal cement is of extreme tenuity in Man, Quadrumana and terrestrial Carnivora; it is thicker in the Herbivora, especially in the complex grinders of the Elephant, and is thickest in the teeth of the Sloths, Megatherioids, Morse, and Cachalot. Vertical folds of enamel and cement penetrate the crown of the tooth in the Ruminants, and in most Rodents, and Pachyderms, characterizing by their various forms the genera of the two last orders : but these folds never converge from equidistant points of the circumference of the crown towards its centre. The teeth of the quadrupeds of the order *Bruta* (*Edentata*, Cuv.) have no true enamel; this is absent, likewise, in the molars of the Dugong, the Zeuglodon and the Cachalot.* The tusks of the Narwhal, Walrus, Elephant, Mastodon, and Dinotherium, consist of modified dentine, which, in the large proboscidian animals, is properly called 'ivory,' and is covered by cement.

"The central part of the fully-formed tooth in man and most other animals contains an irregular kind of osseous substance, which is most abundant in the Cachalot, and forms around foreign bodies which may gain admission to the pulp-cavity of tusks. A fifth substance which, from the number and regular position of the vascular canals in it, I have termed the 'vascular dentine,' forms the body or axis of the tooth in the Sloth-tribe, and is present in smaller proportion in the centre of the teeth of the Armadillos. The teeth of the Orycteropus consist of congeries of long and slender prismatic columnar denticles, each consisting of a body of dentine, with a coating of cement, by which they are united together to form a composite tooth, as in some of the Cartilaginous Fishes." P. 302.

The general conditions of the structure and of the development of the Mammalian teeth, have been already treated of in the Introductory portion of the work. "In most of this class the body of the tooth consists of a

---

* "M. Fr. Cuvier divides the teeth of Mammalia, according to their composition, into four classes : the first consist of ivory, (dentine,) enamel and cement; the second of ivory and enamel; the third of ivory and cement; the fourth of ivory only. '*Dents des Mammifères*,' p. xxi. I have met with no Mammalian teeth, in which cement is absent, and believe that the second and fourth of the above-cited classes of teeth, have no existence in Nature."

gelatinous animal basis, and calcareous earth, combined and arranged according to the plan which characterises the tissue called 'unvascular dentine.'" But "in the incisors of certain Rodents, and in the molars of the Sloth and Megatherioids, or the large extinct phyllophagous *Bruta*, more or less of the dentine is modified by the persistence of certain tracts of the pulp-cavity, forming vascular or medullary canals;" in other words, "vascular dentine."

We have seen, in the fishes, that in many species the development of the matrix of the tooth has been arrested at its first or papillary stage; that in the Reptilia it advances another step; but it is only in the present class that, in all cases, "the matrix sinks into a furrow and soon becomes inclosed in a cell in the substance of the jaw-bone from which the crown of the growing tooth extricates itself by exciting the absorbent-process, whilst the cell is deepened by the same process and by the growth of the jaw, into an alveolus for the root of the tooth. When the formative parts of the tooth are reproduced indefinitely to repair by their progressive calcification the waste to which the working surface of the crown of the tooth has been subject, the alveolus is of unusual depth, and of the same form and diameter throughout, except in the immature animal when it widens to its base. In teeth of limited growth, the dentinal pulp is reproduced in progressively decreasing quantity after the completion of the exterior wall of the crown, and forms by its calcification one or more roots or fangs, which taper more or less rapidly to their free extremity. The alveolus is closely moulded upon the implanted part of the tooth; and it is worthy of special remark that the complicated form of socket which results from the development of two or more fangs is peculiar to animals of the class Mammalia."*

The formation of the second tooth from the matrix of the predecessor, is a theory asserted by all previous authors since the time of Hunter; and it takes place in most of the mammalia. In some cases the new tooth takes the place of its predecessor; in others, it takes its situation by its side. The process by which this formation of a second tooth originates is properly termed gemmiparous, and certainly not, as Professor Owen calls it, "fissiparous."

In entering on the consideration of the particular groups of the class, the horny teeth of the ornithorynchus and echidna are described; and these are followed by an elaborate examination of the structure of the palatine substitutes for teeth in the true whales. Treating these curious appendages as modifications of teeth, the descriptions are adapted to this view of their nature. "The baleen-pulp is situated in a cavity at the base of the plate, like the pulp of a true tooth; whilst the external ce-

---

* "On the strength of this generalization I have established the Mammalian nature of the huge extinct animal called *Basilosaurus* by Dr. Harlan, and have advocated the claims of the diminutive *Amphitherium* and *Phascolotherium* of the oolite slate of Stonesfield, to be admitted into the same high class against the objections raised by Dr. de Blainville. See Comptes Rendus de l'Acad. des Sciences, Oct. 22, 1838. The bifid base of the teeth of certain Sharks not being implanted in a socket, forms no true exception to the rule enunciated in the text. Geological Transactions, 2nd Series, vol. vi. p. 66.

menting material maintains, both with respect to this pulp, and to the portion of the baleen-plate which it develops, the same relations as the dental capsule bears to the tooth. According to these analogies, it must follow, that only the central fibrous or tubular portion of the baleen-plate is formed, like the dentine, by the basal pulp, and that the base of the plate is not only fixed in its place by the cementing substance or capsule, but must also receive an accession of horny material from it, as Hunter first indicated; this material answers to the cement of true teeth."

The description of the baleen-plates by Hunter, introduced in the following passage, is very characteristic of his homely but striking and graphic and truthful style.

"Each plate of baleen consists of a central coarse fibrous substance, and an exterior compact fibrous layer: but this reaches to a certain extent only, beyond which the central part projects in the form of the fringe of bristles. John Hunter first expressed a belief, that the part of the baleen, which was formed by the core or pulp was the hair: and that this received the compact layers on the outside, from the dense but vascular substance between the bases of the plates; which substance he well describes to act as abutments to the whalebone, like the alveolar processes of the teeth, keeping them firm in their places. And he further observes: 'As both the whalebone and intermediate substance are constantly growing, and as we must suppose a determined length, necessary, a regular mode of decay must be established, not depending entirely on chance, or the use it is put to. In its growth three parts appear to be formed; one from the rising core, which is the centre; a second on the outside; and a third being the intermediate substance. These appear to have three stages of duration; for that which forms on the core, I believe, makes the hair, and that on the outside makes principally the plate of whalebone; this, when got a certain length breaks off, leaving the hair projecting, becoming at the termination very brittle; and the third or intermediate substance, by the time it rises as high as the edge of the skin of the jaw, decays and softens away like the old cuticle of the sole of the foot when steeped in water.'

"A thin transverse section of baleen, viewed with a low magnifying power, demonstrates that the coarse fibres, as they seem to the naked eye, which form the central substance, are hollow tubes, with concentric laminated walls. When a high magnifying power is applied to such a section, the concentric lines are shown not to be uniform; but interrupted here and there by minute elliptical dilatations, which are commonly more opake than the surrounding substance, and which, like the calcigerous cells of true bone, are probably remains of the primitive cells of the formative substance; similar long elliptical opake bodies or cells, are dispersed irregularly through the straight parallel fibres of the dense outer laminæ of the baleen plate.

"The chemical basis of baleen, according to Brande, is albumen hardened by a small proportion of phosphate of lime." P. 316.

We have hitherto confined our remarks and extracts to the generalities with which each chapter commences, because it would be impossible, without some such restriction, to attempt anything like an analysis of the work, within a moderate space. The subject of Human Dentition, however, is so important, and so elaborately treated in the present work, that we shall deviate from the rule which we have prescribed to ourselves, and enter somewhat at large into this subject. The general structure and arrangement of the teeth in Bimana, are similar to those of the highest forms of Quadrumana; but there are still some interesting and important distinctions in both particulars.

The researches of Mr. Owen on the history of the larger Apes, are already well known to all zoologists. By them he has been enabled to overcome the difficulties arising from the imperfect accounts which have from time to time been given of these animals; and which have been so various as to have rendered the distinction of the species of this group altogether a matter of ambiguous speculation; whilst at the same time a want of more complete information on the one hand, and, on the other, perhaps an imperfect or erroneous application of the information actually possessed, had tended to perpetuate much of the obscurity. In the papers of Professor Owen in the Zoological Transactions, the characters of the teeth at different ages, in both the genera of the large anthropomorphous apes are brought to bear in an admirable manner upon these points, and exhibit a remarkable instance of clear and acute scientific criticism. We were therefore prepared for the full and satisfactory account which we find in the present work of the teeth of these animals, and which is as complete in an anatomical point of view, as the former treatise was in relation to formal Zoology. Omitting the long and elaborate account of the forms, situation or position, and succession, we proceed to quote the concluding passage of the detail of the structure, which brings us to the account of the teeth of the human subject.

"The microscopic structure of the teeth of the Quadrumana is conformable throughout the platyrhine and catarhine groups, and closely resembles that which Purkinje, Retzius, Muller, and myself have described in the Human subject. As this structure will be more particularly elucidated in the chapter on the Human teeth, I shall here merely mention the most obvious differences which the teeth of the Quadrumana present, and which are manifested not only in the Baboons and Monkeys, but in the most anthropoid ape, viz. the Chimpanzee. In the incisor and canine the general direction of the calcigerous tubes agrees with that figured in Fraenkel's Thesis, and by Retzius in his "Mikroskopiska undersokningar,' in the corresponding Human teeth. The tubes in the Chimpanzee describe the same primary curvatures, but less strongly; and the secondary gyrations are longer and more feebly marked: they are $\frac{1}{10000}$th of an inch in diameter, and the interspace between two tubes on the same plane equals the width of two tubes, when viewed in transverse section near the pulp-cavity; nearer the enamel the interspaces are wider; but in general they are more closely arranged and relatively more numerous, besides being straighter than in the Human subject. They maintain the same diameter through three-fourths of their course; divide sparingly, except close to the enamel boundary and the periphery of the fang: in this part of the tooth the minute lateral branches are most numerous. The calcigerous cells of the dentine are most conspicuous, as usual, near the periphery of the crown, where their well-defined semi-circular contour is seen with its convexity next the enamel; from ten to twelve dentinal tubes on the same plane are included in the diameter of a peripheral calcigerous cell: the cells decrease in size and increase in number, and become less definable as they become situated nearer the pulpy cavity. The enamel fibres are more wavy, even, than in the human teeth: they are $\frac{1}{6000}$th of an inch in thickness, and manifest the striated indication of their cellular origin as distinctly as in the human teeth. The vertical fibres ascending from the summit of the crown of the canine of the Chimpanzee described twenty acute-angled undulations in their course. In the section examined, the bend in one direction transmitted more light than in the opposite, and gave an appearance of waves upon the cut surface of the enamel. This is the character attempted to be shown by Fraenkel in fig. 4 of his thesis, and which Retzius says he had not succeeded in observing in human enamel. The lines of growth or strata of the enamel were best displayed in transverse sections of the incisors and canines. The cement is thickest at the apex of the fang; the Purkinjian cells are traceable to near the neck of the tooth, over which the clear basis of the cement is continued upon the enamel: the tubuli continued from the cells are most numerous on the side next the dentine: they form likewise rich anastomotic plexuses in the interspaces of the cells, besides communicating with peripheral ramifications of the tubuli of the dentine: their diameter is $\frac{1}{15000}$th of an inch." P. 451.

Thus then, having reached in the Chimpanzee the highest step in the series of the brute creation, "our succeeding survey of the Human dental system, expanded by retrospective comparisons, becomes fraught with peculiar interest, since every difference so detected establishes the true and essential characteristics of that part of Man's frame."

"The most marked distinction between the dentition of Man and that of the highest Quadrumanes is the absence of the interval between the upper lateral incisor and the canine, and the comparatively small size of the latter tooth: but its true character is indicated by the conical form of the crown, which terminates in an obtuse point, is convex outwards and flat or sub-concave within, at the base of which surface there is a feeble prominence: the conical form I find best expressed in the Melanian races, especially the Australian: the canine is more deeply implanted, and by a stronger fang than the incisors; but the contrast with the Chimpanzee is sufficiently manifest. There is no sexual superiority of size, either of the canine or any other single tooth in the human subject." P. 453.

The author then proceeds to describe more fully the distinction between the teeth of Man and the higher Quadrumana in detail, especially with regard to the deciduous series; and concludes, with reference to these, that "the differences brought out by the foregoing comparisons, though less striking than those exhibited by the permanent teeth, will be appreciated by the philosophical anatomist as yielding more certain evidence of the essential distinction of the Bimanous species: he will perceive that they are not due to mere adaptive developments, but are manifested at a period when the subjects of comparison are far from having attained the pre-ordained term of deviation from the common primordial type, and antecedent to those changes in the dental system itself, which more broadly characterise the species, and, in the Orang and Chimpanzee, proceed further to mark the different sexes."

The general statement of the component parts of the human tooth is thus given: "The body of the tooth consists of hard or unvascular dentine; the exserted part or crown is invested by enamel; the whole tooth is coated by cement, but this attains the thickness requisite for the development of its characteristic radiated cells only upon the fang or fangs. Every tooth has an internal cavity, which contains the remains of the vascular dentinal pulp, and is thence termed 'pulp-cavity': it is progressively diminished in size as the dentine is completed; remains widest at the base of the crown, and contracts as it descends along the fang or fangs, dividing according to the number of these; and, when they are connate and form apparently a simple root, the pulp-cavity indicates by its division into linear fissures, continued from the coronal dilatation, the number of fangs which compose such undivided root. After the pulp-cavity has been reduced by the completion of the dentine, it is further diminished by the conversion of part of the residuary pulp into a layer of osseo-dentine.

The cement, which is thickest at the end of the fangs, sometimes penetrates the pulp-cavity, and blending with the osseo-dentine closes the aperture, except where two or more minute canals perforate it for the passage of capillary bloodvessels, and nerve filaments."——Now, in reference to the said "Osseo-dentine" we must confess ourselves at a loss to discover where is the essential distinction, excepting that difference which arises from change of circumstances, between this substance and the normal dentine. It is the result of the "conversion," to use our author's theory, or "secretion," according to others, of the same substance as originally produced the latter substance, and differs only in some minute characters of its structure; we should, therefore, certainly consider it more strictly correct to view it in the light of modified dentine, than as a substance deserving a distinct appellation, and viewed as a fourth component of the human tooth. A more particular account of the author's ideas on the subject of this peculiar substance is given farther on. "In my report to the British Association in 1838, which contains the first announcement of some of the observations described in detail in the present work, I stated, with respect to the component structures of a tooth, 'that in addition to those usually described and admitted, there were other substances entering into the composition of teeth, and presenting microscopic characters equally distinct both from ivory, enamel, and cement, and from true bone, and as easily recognisable.'* Of these is the tissue, there first defined, and which I have since called 'osteo-dentine,' from its combining the vascular concentric-coated canals of the osseous tissue, with a development of the fine tubes resembling those of true dentine, but with stronger and less regular curvatures. This substance is found lining the pulp-cavity of old teeth; and sometimes forms the middle part of the grinding surface of much worn molars; but the conversion of the remains of the pulp into the osteo-dentine is constant in human teeth after the age of twenty years.†"——Now, the view which we should

---

* "One of these substances is characterized by being traversed throughout by numerous coarse canals, filled with a highly vascular medulla or pulp, sometimes anastomosing reticularly—sometimes diverging and frequently branching, —sometimes disposed nearly parallel with one another, and presenting more or fewer dichotamous divisions. The canals in many cases are surrounded by concentric lamellæ, and thus resemble very closely the Haversian canals of true bone; but the calcigerous tubes which everywhere radiate from them are relatively much larger. The highly-organized tooth-substance just described differs from true osseous substance, and from cœmentum, in the absence of the Purkinjian corpuscles or cells."—Trans. Brit. Assoc. 1838. p. 137.

† Lintott 'On the Structure of the Human Teeth,' 1843. John Hunter appears first to have called attention to this process, which prevents the exposure of the pulp cavity when the crown is worn low down; he calls the substance 'new matter,' and says 'it may be easily known from the old, for when a tooth has been worn down almost to the neck a spot may always be seen in the middle, which is more transparent, and at the same time of a darker colour, and generally softer than the other.' Hunter's success in injecting vessels in the cavities of the teeth in very old people is explained by the existence of vascular canals in the osteo-dentine and their communication with similar canals perforating the thickened cement. I have already noticed the abundance of this substance in the teeth of the Cetacia, and its centrifugal development from detached centres be disposed to take of this structure is this; that, as soon as the tooth has received its essential form and solidity of structure, the normal office of the pulp ceases; but to provide against further injury, from wearing or other causes of loss of substance, the pulp is still endued with a continued power of producing additional internal portions of solid matter, differing from its normal product only by certain modifications of intimate structure and density. Hence arises the fact, that in the teeth of sailors and others who have been accustomed for years to masticate food which requires very severe trituration, the teeth become worn down until the internal pulp-cavity would be inevitably opened, were it not that it becomes filled up gradually by a fresh "deposit" from, or "conversion" of the residuary pulp in a substance which differs chiefly from the normal dentine in its greater transparency, its more yellow hue, the less proportion of earthy matter, and in certain modifications of its intimate structure. There are some remarkable instances on record of such a filling up of the pulp-cavity by this modified dentine, the transparency of which has been so perfect as scarcely to allow of the possibility of any distinct earthy deposits existing within its substance.

"Of the four substances composing the human tooth that which is most exposed to outward influences, and which is first to operate upon the food in preparing it for deglutition, is the hardest, the most solid, the least organized. We have seen that every fibre of the enamel is so disposed, by the preliminary disposition of the cells in which it was moulded, as to give it the utmost strength and power of resistance of which such a tissue could be capable. The polished surface, the pearl-white colour of this dense and brittle substance, adds ornament to use. In the second and principal substance of the tooth, the dentine, we have traced an equally beautiful arrangement of the earthy salts in directions which best resist both vertical and lateral pressure, but with the additional economy of the substitution of the hollow column for the solid prism. The saving of material is however the least of the benefits gained by this tubular structure of the dentine: the vitality of the tissue, which Hunter recognized so forcibly, but which, being equally convinced of the non-vascularity of the tissue, he was unable to explain,—willing rather to enunciate an apparent paradox, or be taunted with dilemma, than yield an iota of either of his convictions,*—is explicable by the possible and highly probable fact of a circulation of the colourless plasma of the blood through the dentinal tubes. That some elementary prolongations of nerve may also be continued into these tubes, who may confidently deny? Whoever has felt the pang produced by contact of a probe with the recently exposed surface of the dentine, must at least allow the tubes to be most efficient conductors of the impression to the sentient pulp. Nature has suffered no part of the dentine to be exposed: its organization and vital powers are adequate to its own support in health, and to control that stimulus which, when the dentine is dead and truly an extraneous body, operates detrimentally upon the surrounding more highly organized parts: but the vitality of the dentine is insufficient for the reproduction of its tissue, or for the arrest or repair of decay. The third

---

in the substance of the pulp of the Cachalot, forming there sometimes detached masses. A modification of osteo-dentine forms, as we have seen, the main body of the teeth of the Sloths and Megatheroid quadrupeds; but the transition from the normal hard dentine to its vascular modification is more gradual in the human teeth."

* "See Prof. Bell's preface to his Edition of 'Hunter on the teeth,' p. xiii."

substance which is chiefly developed around the implanted part of the tooth is more highly organized than the dentine, more analogous to bone, and accordingly better adapted for vital connexion with the vascular membrane of the alveolus. It is the cement which renders a living tooth capable of uniting with any part of a living body, as Hunter proved by his ingenious experiments." 469-71.

This is an admirable digest of the comparative characters and offices of the various parts of which a tooth is composed. It may, however, admit of some doubt whether the calcigerous tubules be the medium by which sensation is conveyed through the substance of the dentine. The non-appearance of nervous filaments, is no proof of the non-existence of nervous function; and if it were, the argument would be equally applicable against the passage of nerves through the tubules. It may be thus; but in the absence of demonstration, or of any strong primâ facie probability, it is, we think, too distinct a proposition, that "whoever has felt the pang produced by contact of a probe with the recently exposed surface of the dentine, *must*, at least, allow the *tubes* to be most efficient conductors of the impression to the sentient pulp." The intertubular substance containing, as it does, the great proportion of animal matter of the tooth, may, with great probability, be the medium of sensation. At least there appears to us no reason in favour of the former proposition.

Mr. Owen maintains the omnivorous character of the human dental apparatus, in opposition to those who consider that the condition of these organs, indicates the frugivorous propensity in man. The following passage offers a strong argument in favour of this view, but whether it be the true theory or not admits, perhaps, of some controversy.

"The Apes and Monkeys which Man most nearly resembles in his dentition, derive their staple food from fruits, grain, the kernels of nuts, and other forms in which the most sapid and nutritious tissues of the vegetable kingdom are elaborated: and the close resemblance between the Quadrumanous and Human dentition shows that Man was, from the beginning, more especially adapted 'to eat of the fruit of the trees of the garden.'*

"But the Quadrumana are not exclusively frugivorous. Some are known to seek the eggs and callow-brood of birds. The African Baboons pass whole hours in turning up great stones in quest of insects. The young Chimpanzees and Orangs in our menageries manifest no repugnance to cooked meat, and the avidity with which they will pluck and devour a sparrow leads us to suspect that their vegetable diet is occasionally varied by the capture of a live bird.

"The formidable development of the canine teeth in the Orangs and Baboons seems, at the first glance, to relate to the predominance of animal food in their regimen; but the sexual difference in the development of those teeth might have indicated that they had other subserviences than to the acquisition of daily sustenance, if observation had not shewn them to have been given to the males for the purpose of combat and defence. The molar teeth are those which form always the best and sometimes the sole guides to a knowledge of the diet of a mammiferous animal: and these clearly indicate the frugivorous and mixed regimen of the Quadrumana and Man.†

* "This is the conclusion to which my friend Prof. Bell has arrived in his 'Physiological Observations on the Natural Food of Man, deduced from the characters of the Teeth.' On the Teeth, 8vo. 1829, p. 33."

† "John Hunter in considering the question, 'Under what class do the

"We have seen that the most striking characteristic of the human dentition is the absence of any disproportionate development of particular teeth, and the concomitant continuity of the series in both jaws: the more immediate comparison with the Quadrumanous dentition, while it demonstrates the close similarity of the molar teeth, and supports the consequent deduction of an omnivorous diet, brings to light the very interesting difference in the proportions and disposition of the incisive and canine teeth, by which we may appreciate the adaptation of the human dentition to Man's peculiar and higher attributes. His reason furnishes him, in the lowest condition of savage life, with weapons more formidable than sharp and long canine teeth, and his mastery over fire, the prime element of cookery, enables him to dispense with strong and prominent incisors in the reduction of the tough, raw, and indigestible parts of vegetable or animal food.

"But the moderate and equable development of the anterior teeth, and the graceful continuous curve which they form with the molar series, have not merely a negative relation to the substitutes which Man's inventive faculty provides for the absence of the large and strong incisors and canines of the Orangs. The smooth and equable posterior surface of the incisors, their vertical position and their close arrangement in a gentle curve with the canines, offer the best conditions for reacting upon the tongue in the various applications of that organ to the teeth during speech. The appearance of the teeth in the mouth is contemporaneous with the child's power of forming articulate sounds; and the clearness of utterance is affected both by the temporary deficiency of the incisive series during the change of dentition, and in a still greater degree by the final loss of the teeth and their sockets in old age.

"Lastly, the vertical symphysis of the human jaw not only affords the most favourable position to the incisors in relation to speech, but is accompanied in the highest races with a prominence of the lower border forming the chin, which is wanting in every inferior animal: and this remarkable feature, with the short jaws proportioned to the small incisors and canines, harmoniously combine with the capacious forehead in stamping the head of Man with the impress of his intellectual superiority." P. 473.

We have turned to the passage in Mr. Bell's work on the teeth, and we confess we see much reason to agree in the opinion there enunciated, that judging "from the formation of his teeth, and digestive organs, as well as from the character of his skin, and the structure of his limbs," "man was originally formed a frugivorous animal, and, therefore, probably, tropical, or nearly so, with regard to his geographical situation."

We cannot pass over, with a mere allusion, the concluding portion of the work, which is devoted to the Proboscidian Pochyderms; for, although it is too long to be transferred to our columns, and too elaborate to be analyzed, yet it exhibits as remarkable examples, as are to be found in the whole work, of that combined philosophical application of truth, and indefatigable search after it, which so remarkably characterize the author's mind. We are reluctantly compelled to limit our extract to two or three passages. The curious and well-known instances, of not very unfrequent occurrence, of balls being found imbedded in the tusks of elephants, are thus accounted for, and the process of their investment by modified ivory elaborately described.

---

Human Teeth come?' concludes by stating, 'He, (Man) ought, therefore, to be considered as a compound, fitted equally to live upon flesh and upon vegetables.' Nat. Hist. of Human Teeth, 4to. 1771, p. 120."

"The central part of the tusk, especially near the base of such as have reached their full size, is occupied by a slender cylindrical track of modified ivory, perforated by a few vascular canals, which is continued to the apex of the tusk. It is not uncommon to find processes of osteo-dentine, or imperfect bone-like ivory, projecting in a stalactitic form into the interior of the pulp-cavity, apparently the consequence of partial inflammation or malformation of the vascular pulp. The musket-balls and other foreign bodies which are occasionally found in ivory, are immediately surrounded by osteo-dentine in greater or less quantity. It has long ceased to be a matter of wonder how such bodies should become completely imbedded in the substance of the tusk, sometimes without any visible aperture, or how a leaden bullet may have become lodged in the solid centre of a very large tusk without having been flattened. Such a ball, aimed at the head of an elephant, may penetrate the thin bony socket and the thinner ivory parietes of the wide conical pulp-cavity occupying the inserted base of the tusk; if the projectile force be then spent the ball gravitates to the opposite and lower side of the pulp-cavity. The presence of the foreign body exciting inflammation of the pulp, an irregular course of calcification ensues, which results in the deposition around the ball of a certain thickness of osteo-dentine. The pulp then resuming its healthy state and functions, coats the surface of the inclosing mass of osteo-dentine, together with the rest of the conical cavity into which that mass projects, with layers of normal ivory. The ivory, as it approaches the osteo-dentine, becomes modified by the presence of vascular canals, and assumes the character of vaso-dentine; and the canals frequently form loops directed towards the osteo-dentine. In this substance the medullary canals diverge, leaving clear interspaces into which numerous irregular branches proceed from the canals, partially uniting to form a net-work, and finally breaking up into distinct cells, of larger size than the Purkinjian cells of the external cement. In the small detached fusiform nodules of osteo-dentine, one or more vascular canals are present with concentric coats of the clear basal substance, studded by Purkinjian cells of the size of those in the cement, but with richer radiating systems of tortuous calcigerous tubes. Some of the larger-sized cells are likewise frequently here present. From both the medully canals, their sub-divisions and the cells, minute calcigerous tubes diverge, with wider intervals and a more wavy and irregular course than the normal tubes of the ivory.

"The portion of the cement forming capsule surrounding the base of the tusk, and the part of the pulp, which were perforated by the ball in its passage, are soon replaced by the active reparative power of these highly vascular bodies. The hole formed by the ball in the base of the tusk is then more or less completely filled up by a thick coat of cement from without and of osteo-dentine from within. Traces of such a cicatrix closing the entrance have been more than once noticed: and Blumenbach deduced, therefrom, a property in the Elephant's tusk to pour out bony matter in order to heal such wounds. The reparation is, however, effected by the calcification of the reproduced parts of the capsule and pulp.

"By the continued progress of growth, the ball so inclosed is carried forwards to the middle of the solidified exserted part of the tusk, as in the example in Blumenbach's collection which he considered so curious. Should the ball have penetrated the base of the tusk of a young elephant it may be carried forwards by the uninterrupted growth of the tusk until that base has become the apex, and be finally exposed and discharged by the continual abrasion to which the apex of the tusk is subjected. Yet none or these phenomena prove the absolute non-vascularity of the tusk, but only the low degree of its vascularity. Blood circulates, slowly no doubt, through the minute vascular canals which are continued through the centre of the ivory to the very apex of the tusk: and it is from this source that the fine tubular structure of the ivory obtains the essential plasmatic colourless fluid by which its low vitality is maintained." P. 645.

Finally, alluding to the difficulties into which Cuvier was driven, as our author says, by his being cramped, as it were, by the excretion theory. He states that this great comparative anatomist "is compelled, by his hypothesis, to deny a matter of fact. The cement (cortical) being, according to him, an excretion from the internal layer of the capsule, could only be present on that part of the teeth over which such layer extended; but the enamel pulp, which passed with Cuvier for the internal layer, unquestionably ceased to exist at the base of the crown or body of the tooth; therefore, Cuvier denies that the fang has any covering of cement."

Now, this may be quite true as regards Cuvier's opinion above stated; but if it be allowed, as already stated by Blake and others, that the cement is produced by the external layer of the sac, or the true capsule, as distinguished from the enamel pulp, the difficulty vanishes, so far as the situation of the formative structure goes, and the two theories are equally compatible with the existing fact. Mr. Owen, however, concludes in these words.

"The conversion theory of dental development as propounded and established in the present Work, leads to no such difficulties: its truth is manifested in the same way as that of the undulation-theory of light, and of every other right theory: viz. by its concordance with facts which are denied or misinterpreted on the wrong theory, and by its more rational explanation of all the phenomena." 655.

Here then we conclude a most imperfect, and unworthy attempt to communicate to our readers, some idea of this extraordinary work; which as the result of united industry, research, ardour in the pursuit of truth, genius in the conception, and clearness in the annunciation of theory is, we believe, without a parallel in the present day. Those only who have gone with careful examination and patient reflection into the details of the work, can have any adequate idea of its fulness and extent, or of the originality and high philosophy of its reasoning. Those, however, who have been acquainted with the previous labours of the distinguished author, will only see in this work, the same high qualities of mind, and the same amazing extent of information, as had already gained for him a fame co-extensive with the spread of science itself. May we not hope that this is but the commencement of a series of monographs upon all the different systems of organs, so that while each would, like the present, be complete in itself, the whole would form such a circle of physiological science, as has never before been attempted, or even imagined. We have heard that such a work has been projected, and we cannot but expres our earnest hope that life and health may be spared to enable him, Mr. Owen, not only to perfect it, but to enjoy for many years after its completion the high and pure gratification of having thus conferred an inestimable boon upon the scientific world.

